# Clinical impact of circulating tumor DNA to track minimal residual disease in colorectal cancer patients. Hopes and limitations

**DOI:** 10.1016/j.esmogo.2024.100068

**Published:** 2024-06-03

**Authors:** C. Soueidy, A. Zaanan, M. Gelli, E. Moati, C. Gallois, V. Taly, P. Laurent-Puig, L. Benhaim, J. Taieb

**Affiliations:** 1 Department of Gastroenterology and Digestive Oncology, Georges-Pompidou European Hospital, Assistance Publique-Hôpitaux de Paris (AP-HP-Paris Centre), Université de Paris, Paris, France; 2 Hematology and Oncology Department, Hotel Dieu de France Hospital, Université Saint Joseph de Beyrouth, Beirut, Lebanon; 3 Centre de Recherche des Cordeliers, INSERM, Sorbonne Université, Université de Paris Cité, CNRS SNC 5096, Paris; 4 Department of Surgery and Visceral Oncology, Gustave Roussy, Cancer Campus, Villejuif, France

**Keywords:** circulating tumor DNA, colon cancer, rectal cancer, adjuvant chemotherapy, minimal residual disease

## Abstract

Circulating tumor DNA (ctDNA) has been studied as a non-invasive tool for disease monitoring in different cancer types. Despite advances in colorectal cancer (CRC) management, it remains a leading cause of mortality and there is an unmet need for new biomarkers to guide therapeutic approaches and improve patient’s outcome after surgical resection of the primary tumor or its metastatic sites. This review summarizes the different clinical results and ongoing studies on the performances of ctDNA as a prognostic marker for disease recurrence, both in non-metastatic patients with resection of the primary tumor and in those with full resection of metastatic disease.

## Highlights

• ctDNA is a strong predictor of recurrence un CRC patients after curative surgery.
• ctDNA is a promising biomarker to select patients with resected colon cancer for adjuvant chemotherapy.
• ctDNA analysis methods should be chosen adequately to fit the goal of a personalized treatment approach.

## Introduction

Colorectal cancer (CRC) is the third most common cancer worldwide in terms of incidence and the second in terms of mortality.^1^ Treatment of early-stage (I-III) colon cancer is based on surgical resection of the tumor with clean margins and appropriate lymphadenectomy. Adjuvant chemotherapy is indicated for all patients with stage III disease and in patients with stage II disease with high-risk features.^2^, ^3^, ^4^ However, it is estimated that 50%-80% of those patients are already cured by surgery.^5^ In addition, in patients with stage IV (metastatic) CRC, there is still a hope for cure if all metastatic lesions can be removed surgically. In all these patients with resectable disease, an appropriate tool to select patients for (pre- and) post-operative chemotherapy is missing.

In both non-metastatic and metastatic CRC (mCRC), the monitoring of disease recurrence once all detectable disease has been removed surgically is based on plasma carcinoembryonic antigen (CEA) assay dosage combined with thoraco-abdomino-pelvic computed tomography scan every 3-6 months for 5 years. However, this surveillance program varies a lot between countries and remains suboptimal in detection of early disease recurrence at a time when curative-intent treatment might still be possible.^6^, ^7^, ^8^ Consequently, a more sensitive and specific tool to identify early recurrence would be of great interest in order to offer to each patient the best therapeutic option.

Minimal residual disease (MRD) is a well-established principle for early identification of persistent disease in many hematological malignancies.^9^ Plasma circulating tumor DNA (ctDNA) could be a new prognostic and predictive tool to detect MRD after CRC surgery, to select patients for adjuvant chemotherapy and to monitor patients more effectively.

This article summarizes the most important studies that have assessed the role of ctDNA in CRC in tracking MRD after surgical resection of the primary tumor or of all metastatic sites and reviews major ongoing trials in this field.

## Circulating tumor DNA

Plasma ctDNA has been studied in multiple cancer types. Due to various mechanisms including cell apoptosis and necrosis or shedding from living cells, cell-free DNA (cfDNA) is released from normal and malignant cells and can be detected in the bloodstream and other body fluids. The ctDNA is the fraction of cfDNA that originates from tumor cells with a half-life of <2 h in the circulation.^10^

The detection and characterization of plasma ctDNA (also called liquid biopsy) is a relevant non-invasive marker that allows for cancer detection and for disease monitoring, potentially overcoming limitations of tissue biopsies. ctDNA analysis can capture the molecular heterogeneity of a patient’s cancer as well as its progression over time. This is especially important in CRC where both spatial and temporal heterogeneity have been described.^11^^,^^12^ Several studies have demonstrated the concordance between ctDNA molecular analysis and tissue analysis with the advantage of shorter turnaround time for ctDNA analysis.^13^^,^^14^

The proportion of ctDNA within total cfDNA varies significantly and depends on multiple factors such as primary tumor location, disease burden, metastatic sites, tumor vasculature, cellular turnover, and pre-analytic conditions.^15^^,^^16^ ctDNA circulates in the form of short fragments with a peak size distribution consistent with the size of DNA wrapped around histones and linker regions (166 bp).^17^, ^18^, ^19^, ^20^ The differences in the size fragmentation features and the epigenetics marks have been proven efficient not only to distinguish ctDNA from non-tumoral circulating DNA but also to define its tissue of origin.

To guarantee the comparability of the results of ctDNA analysis, the pre-analytical conditions must be carefully considered. Even though there is no definitive consensus on the appropriate pre-analytical conditions, specific points have been described as important by a wide range of studies. These considerations include the use of dedicated tubes that stabilize nucleated blood cells, such as Streck (Streck (R), La Vista, Nebraska) cfDNA BCT tubes, to avoid contamination by long-sized DNA fragments (and thus dilution of its tumor fraction), and to allow for sample stabilization at room temperature for several days.^21^^,^^22^ Two centrifugations steps for plasma preparation and plasma storage at −80°C are needed to ensure pertinent results. Moreover, plasma samples should be preferred to serum samples to avoid contamination by long DNA fragments.^23^, ^24^, ^25^

In addition, the sampling schedule should be adapted to the clinical question addressed. It is indeed known that cfDNA levels can vary in responses to several pathological and non-pathological conditions, including surgical trauma.^26^, ^27^, ^28^, ^29^, ^30^, ^31^, ^32^, ^33^, ^34^ Repeated ctDNA testing at a distance from surgical resection for patients with early negative detection can also be considered.

Recent technological advances especially suited to liquid biopsies have greatly facilitated the analysis of ctDNA and largely contributed to the increased demonstration of its utility.^35^ However, as mentioned by a recent European Society for Medical Oncology (ESMO) recommendations, ‘there are no single ctDNA assays that would be fit for all purposes’.^21^ The targeted clinical application should drive the choice of ctDNA detection methods since they present differences related to their sensitivity, cost, and turnaround time.

These strategies include the analysis of a single or limited number of alterations using, for example, PCR-based procedures such as digital PCR (dPCR) or the analysis of many alterations using next-generation sequencing (NGS)-based targeted panels, allowing the analysis of hundreds of alterations or genome-wide analysis. Targeting a large number of alterations tends to increase sensitivity in detection of ctDNA.^36^

dPCR relies on the use of microcompartments in the form of microdroplets [(droplet-based digital PCR (ddPCR)] or microchambers to partition the sample for testing using two- to six-color systems. It allows for high-precision ctDNA detection and quantification and presents high sensitivity (<0.01%). It generally has a turnover time of a few days which is particularly relevant in the adjuvant setting.^37^^,^^38^ Other quantitative PCR-based mutation detection methods allowing for highly sensitive detection have also been described.^39^^,^^40^ Approaches employing massively parallel sequencing and optimized library preparation techniques for ctDNA detection have also been largely described.^41^ These approaches include targeted amplicon sequencing [i.e. Safe Sequencing System (Safe SeqS)], tagged-amplicon deep sequencing (TAm-Seq), and hybrid capture-based NGS methods [i.e. cancer-personalized profiling by deep sequencing (CAPP-seq)] and their optimizations.^42^, ^43^, ^44^ Other highly sensitive optimized NGS methods have been studied using different strategies to reduce sequencing artifacts based on the use of molecular barcoding and/or bioinformatics.^42^^,^^43^^,^^45^, ^46^, ^47^, ^48^ These strategies allow for more accurate and precise mutation identification and quantification of mutations, making them more appropriate for MRD applications than conventional targeted amplicon NGS-based methods. They have a very low limit of detection, which allows detection of variant allele frequencies <0.01, and can detect high numbers of specific mutations, including deletions, rearrangements, and copy number alterations. Moreover, by allowing the analysis of multiple loci, NGS-based methods could lead to higher sensitivity for ctDNA detection than methods with lower multiplexing abilities. The main disadvantages of these tests are their relatively high cost, complex analysis procedures, and their long turnover time, which is a drawback when there is an urgent need for a treatment decision, as in the adjuvant setting.^41^

**Figure 1:**
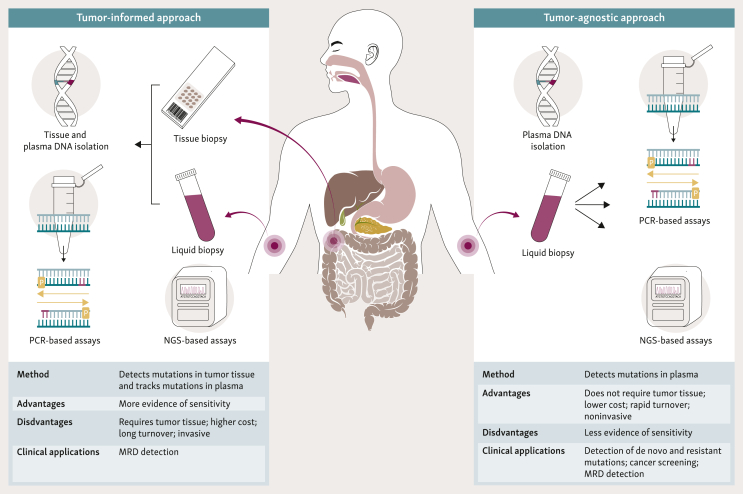
**Tumor-informed approach versus tumor-agnostic approach to determination of ctDNA.** ctDNA, circulating tumor DNA; MRD, minimal residual disease; NGS, next-generation sequencing.

Two main approaches are used to detect ctDNA in the context of MRD assessment: tumor-informed and tumor-agnostic. Their main characteristics are summarized in Figure 1. Tumor-informed strategies are based on prior characterization of specific alterations present in the primary tumor. These alterations can be determined using several approaches including whole genome or exome sequencing, which can detect genomic modifications, but also epigenetic changes, allowing the tracking of multiple cancer-specific mutations simultaneously. Other methods can be applied to the characterization of the primary tumor, including looking into smaller mutation panels that include the most frequent alterations. Digital PCR and/or personalized NGS-based assays could be the methods of choice for the subsequent detection of patient-specific alterations in plasma. Personalized monitoring algorithms that track alterations of interest from non-personalized sequencing panels have also recently been described.^49^^,^^50^ These personalized approaches present high accuracy for ctDNA analysis, but generally have long turnaround times and elevated costs.

Tumor-agnostic approaches do not require previous knowledge of the tumor and directly test the plasma. A large range of techniques have been developed for this approach based either on the parallel testing of many alterations (either targeted or genome-wide) or on assays focusing on a restricted panel of markers allowing for ‘universal’ detection of ctDNA.^18^^,^^20^^,^^51^ Several approaches rely on specific high-sensitivity optimized NGS that uses bioinformatic pipelines and targeted gene panels containing frequently mutated genes in a specific disease, such as BRAF, RAS, TP53, and APC for CRC.^47^ Agnostic approaches present the obvious advantages of bypassing the need for tumor tissue, thus leading to a shorter turnover time and potentially reduced cost. The integration of other markers such as methylation tends to increase the sensitivity of tumor-agnostic tests. Other strategies based on fragmentomics and/or multimodal analysis have also been described for MRD, but most of the published data were focused on early cancer detection.^10^, ^11^, ^12^^,^^15^^,^^16^^,^^52^, ^53^, ^54^, ^55^

## Circulating tumor DNA to predict recurrence after curative surgery and guide adjuvant therapy

### Non-metastatic colon cancer

Several studies have tested the ability of ctDNA to detect MRD and to predict early recurrence after surgery and after adjuvant chemotherapy in non-metastatic colon cancer patients (Table 1). ctDNA was detected post-operatively in 7%-25% of patients, depending on disease stage and publications.^56^, ^57^, ^58^, ^59^, ^60^, ^61^, ^62^, ^63^ Patients with detectable ctDNA after surgery have a higher risk of recurrence compared with patients with negative post-operative ctDNA with a hazard ratio (HR) ranging in the current literature from 1.8 to 18.^56^, ^57^, ^58^, ^59^, ^60^, ^61^, ^62^, ^63^ In addition, ctDNA was considered as an independent prognostic marker in most of these studies after adjustment of the most important, currently known, prognostic factors in non-metastatic colon cancer (Table 1).

**Table 1:** Cohorts and post hoc analyses evaluating the role of ctDNA in predicting recurrence after surgery and adjuvant chemotherapy in non-metastatic resected colorectal cancer

Study	Pathology and stage	ctDNA analysis	Sample size	% of positive ctDNA	Results
Tie et al.^61^	Colon stage II	Safe-SeqS	230	Post-op: 7.9% Post-chemo: 11%	- Post-op: 3-year RFS 0% in ctDNA+ versus 90% in ctDNA− (HR 18) - Post-chemo: 3-year RFS 0% in ctDNA+ versus 85% in ctDNA− (HR 11)
Tie et al.^62^	Colon stage III	Safe-SeqS	96	Post-op: 20% Post-chemo: 17%	- Post-op: 3-year RFS 47% in ctDNA+ versus 76% in ctDNA− (HR 3.8) - Post-chemo: 3-year RFS 30% in ctDNA+ versus 77% in ctDNA− (HR 6.8)
Reinert et al.^56^	Colorectal stage I-III	Multiplex PCR and NGS	130	Post-op: 10.6% Post-chemo: 8%	- Higher risk of recurrence in post-op ctDNA+ (HR 7.2), in post-chemo ctDNA+ (HR 17.5) and during surveillance (HR 43.5) - Serial ctDNA can detect MRD up to 16.5 months earlier than radiologic imaging
Wang et al.^59^	Colorectal stage I-III	Safe-SeqS	58	27.5% of patients who did not receive chemo 11% of patients who received chemo	- All patients who had disease recurrence were ctDNA+ - All patients who had no recurrence after chemo were ctDNA– - 3/32 patients who did not receive chemo and had recurrence were ctDNA+ - ctDNA positivity preceded radiologic and clinical recurrence by a median of 3 months
Tarazona et al.^58^	Colorectal stage I-III	Multiplex PCR	193	Post-op: 9.2%	- Higher recurrence in post-op ctDNA+ (HR 16.53), post-chemo (HR 27.92) and after definitive treatment (HR 47.52) - Inferior RFS in ctDNA+ (HR 53.19) - Serial ctDNA can detect MRD 9.08 months earlier than radiologic imaging
Benhaim et al.^57^	Colorectal stage II-III	ddPCR	184	Preop: 27.5% Post-op: 10.5% Long-term follow-up: 15%	- Recurrence rate 32.7% in preop ctDNA+ versus 11.6% in ctDNA– - Shorter TTR in ctDNA+ preop (HR 3.58), immediately after surgery (HR 3.22) and 1-6 months post-op (HR 5) - ctDNA can detect recurrence 13.1 months earlier than radiologic imaging
Henriksen et al.^63^	Colorectal stage III	Multiplex PCR, NGS	168	Post-op: 14% Post-chemo: 10.7%	- Post-op ctDNA+ was a recurrence predictor (HR 7) - Post-chemo ctDNA+ was a recurrence predictor (HR 50.76) - Recurrence rate in post-op ctDNA+ 80% - All patients with post-chemo ctDNA+ had relapse - Serial ctDNA was a recurrence predictor (HR 50.80) - ctDNA growth rate was prognostic of survival (HR 2.7) - ctDNA detected recurrence with a median lead time of 9.8 months compared to SOC imaging
Taieb et al.^67^	Colorectal stage III	ddPCR for methylation markers WIF and NPY	1017	Post-op: 13.8%	- 3-year DFS 66.39% in ctDNA+ versus 76.71% in ctDNA− (*P* = 0.015) - ctDNA is an independent prognostic factor for DFS (HR 1.55) and OS (HR 1.65) - ctDNA+ patients seem to undergo 6 months of adjuvant chemotherapy as compared to 3 months
Grancher et al.^64^	Colon stage II	ddPCR	134	Post-op: 9%	- ctDNA was detected more post-op in recurrent group (16.7% versus 1.8%, *P* = 0.002) - DFS 16.8 months in ctDNA+ versus 54 months in ctDNA− (*P* = 0.002) - OS 51.3 months in ctDNA+ versus 69.5 months in ctDNA− (*P* = 0.03) - ctDNA was associated with recurrence (OR = 11.13, *P* = 0.03) and death (HR = 3.15, *P* = 0.01)
Mo et al.^60^	Colorectal stage I-III	Multiplex PCR for ctDNA methylation markers	296	Preop: 78.4% Post-op: 23% Post-chemo: 12%	- Higher risk of recurrence in post-op ctDNA+ versus ctDNA− (HR 17.5) - The integration of ctDNA and CEA showed risk stratification for recurrence (HR 19) - Shorter RFS in post-chemo ctDNA + versus ctDNA– (HR 13.8) - Shorter RFS in post-definitive treatment ctDNA+ versus ctDNA− (HR 20.6)

CEA, carcinoembryonic antigen; ctDNA, circulating tumor DNA; DFS, disease-free survival; ddPCR, droplet-based digital polymerase chain reaction; HR, hazard ratio; RFS, recurrence-free survival; MRD, minimal residual disease; NGS, next-generation sequencing; OR, odds ratio; OS, overall survival; post-op, post-operative; preop, preoperative; SOC, standard of care; TTR, time to recurrence.

Post-operative positive ctDNA was associated with worse recurrence-free survival (RFS) in both stage II and III resected colon cancer, in the first publications on this topic by Tie et al. 7 years ago^61^^,^^62^ (Table 1). More recently, a case-control study including 134 patients with stage II resected colon cancer from the PRODIGE 13 trial showed not only better progression-free survival (PFS) in patients with post-operative negative ctDNA, but also better overall survival (OS) compared to positive ctDNA patients.^64^ In addition, ctDNA positivity after adjuvant chemotherapy was also predictive of worse outcomes with increased risk of disease recurrence and a shorter RFS compared to ctDNA-negative patients in several reports^56^^,^^58^, ^59^, ^60^, ^61^, ^62^, ^63^ (Table 1).

Two recent meta-analyses, including 6 and 37 studies, respectively, also demonstrated the prognostic value of ctDNA after surgery and adjuvant treatment.^65^^,^^66^ Moreover, different studies have shown the ability of serial ctDNA testing after surgery to detect MRD earlier than with radiologic imaging, with intervals ranging from 1 to >16.5 months (Table 1).

Despite these interesting results, the previously mentioned studies have some limitations including suboptimal pre-analytic conditions, limited number of ctDNA-positive patients, mixing stage I-III disease, limited follow-up time, limited information on adjuvant treatments, and lack of multivariable analyses adjusting for all relevant confounding prognostic factors. Therefore, they need to be supported by large prospective studies or clinical trials with pre-defined objectives to confirm their results.

A first large series, from a randomized phase III trial, came with the *post hoc* analysis from the PRODIGE-GERCOR IDEA-FRANCE trial. This study analyzed ctDNA of 1017 patients with resected stage III colon cancer treated with oxaliplatin-based adjuvant chemotherapy, using two methylation markers: WIF and NPY by ddPCR. At a median follow-up of 6.6 years, the 3-year disease-free survival (DFS) was worse in patients with post-operative positive ctDNA compared to patients with negative ctDNA (66.39% versus 76.71%). ctDNA was an independent prognostic marker for DFS and OS. Finally, ctDNA-positive patients seemed to undergo 6 months of adjuvant chemotherapy as compared to 3 months^67^ (Table 1). However, pre-analytical conditions were not optimal in this work.

**Figure 2:**
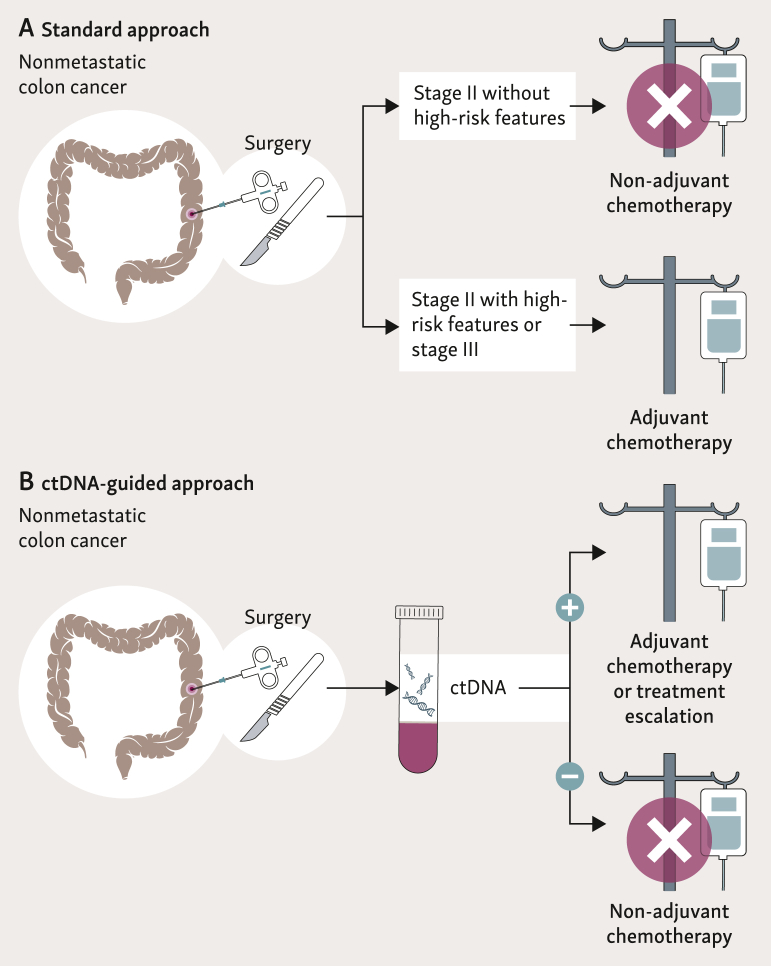
**Standard versus ctDNA-guided approach to prescription of adjuvant chemotherapy.** ctDNA, circulating tumor DNA.

Following these interesting results, many ongoing clinical trials are evaluating the role of ctDNA in prediction of disease recurrence and in guidance of treatment decision making in patients with fully resected primary disease (Figure 2).

**Figure 3:**
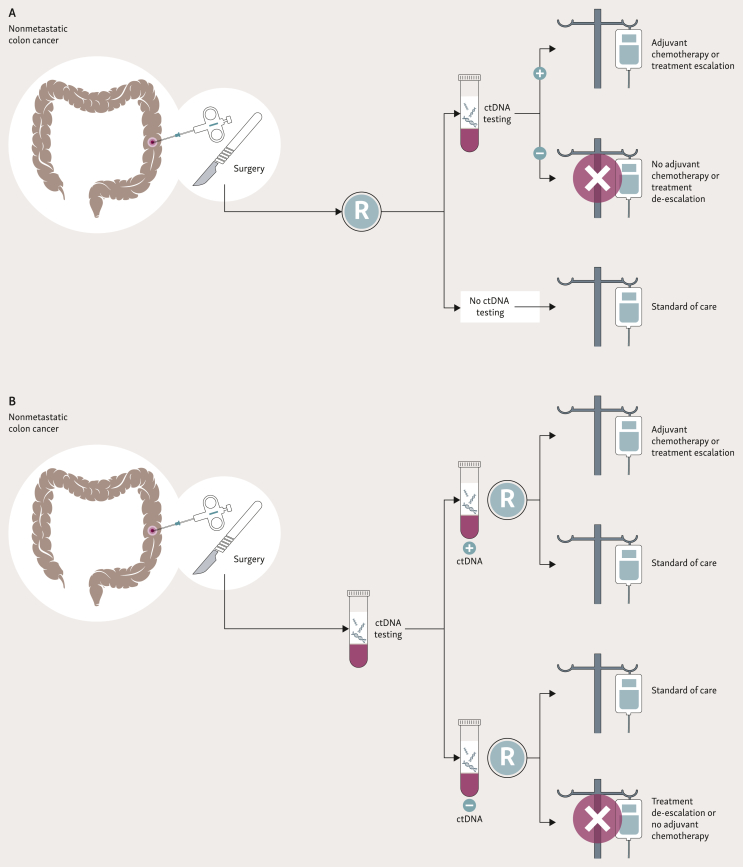
**Randomization of both ctDNA− and ctDNA+ patients to test innovative treatment options (escalation, de-escalation, omission, targeted agents).** (A) Trials testing the added value of ctDNA testing, ‘testing the test’. (B) Trials testing new treatment approaches based on ctDNA, ‘testing treatment options’. ctDNA, circulating tumor DNA.

On the one hand, trials have been designed to test a ctDNA-informed therapeutic strategy versus standard of care without ctDNA testing, in other words trials that are ‘testing the test’ (Figure 3A).

The randomized clinical trials DYNAMIC II and III evaluated the outcome of patients with stage II and III resected colon cancer using a ctDNA-guided management compared to the standard approach, in terms of prescription of adjuvant chemotherapy. The DYNAMIC II trial was recently published.^68^ This trial included 455 patients with stage II colon cancer, randomized 2 : 1 for a ctDNA-guided or standard treatment. Final results showed that 15% of patients in the ctDNA-guided management group received adjuvant chemotherapy versus 28% in the classical management group based on clinicopathologic features. ctDNA-guided management was not inferior to the standard approach in terms of 3-year RFS (91.7% versus 92.5%) and in 2-year disease recurrence rates. In addition, the 3-year RFS was 86.4% in ctDNA-positive patients who received chemotherapy and 92.5% in ctDNA-negative patients who did not receive chemotherapy. This study showed that a ctDNA-guided approach could reduce the use of adjuvant chemotherapy for patients with stage II disease without affecting RFS. However, although these results are impressive and pragmatic in a population-based treatment approach, they also show that the chemotherapy regimens differed between the two trial arms, with 62% of oxaliplatin-based chemotherapy in the ctDNA group and 90% of single-agent fluoropyrimidine in the control group, thereby possibly impacting the global study results. A second limitation is that in ctDNA-negative patients with resection of a T4 tumor, the recurrence rate was as bad as in ctDNA-positive patients, underlining the fact that ctDNA is not sufficient to fully assess the risk of disease recurrence in stage II colon cancer patients from an individual-based treatment perspective (Table 2) (Figure 3A). The DYNAMIC III trial is currently enrolling patients.

**Table 2:** Clinical trials evaluating the role of ctDNA in predicting recurrence after surgery ± adjuvant chemotherapy in non-metastatic resected colon cancer

Trial name	Pathology and stage	Arms	ctDNA analysis	Sample size	Primary endpoint	Preliminary results
DYNAMIC II^68^	Colon stage II	ctDNA-guided group versus standard group	Safe-SeqS	455	2-year RFS	- 15% in the ctDNA group versus 28% in the standard group received chemo - 2-year RFS was not inferior in the ctDNA group to the standard group (93.5% and 92.4%) - 3-year RFS 86.4% in ctDNA+ versus 92.5% in ctDNA−
DYNAMIC III 2022	Colon stage III	ctDNA-guided group versus standard group	Safe-SeqS	1000	RFS	Not available
TRACC^69^^,^^70^	Colon high-risk stage II and stage III	ctDNA-guided group versus standard group	Signatera	107	- Incidence of ctDNA+ before surgery - Post-op ctDNA correlation with DFS	- 93.4% ctDNA+ before surgery - 13% MRD+ after surgery - 42.9% of MRD+ relapsed versus 8.6% MRD− (HR 10) - ctDNA prognostic factor associated with RFS (HR 28.8)
MEDOCC-CrEATE^71^	Colon stage II	ctDNA-guided group versus standard group	PGDx elio™	1320	Proportion of patients receiving adjuvant chemo when ctDNA+ after surgery	Not available
COBRA^72^	Colon stage IIA	ctDNA-guided group versus standard group	LUNAR®	1408	- ctDNA clearance with or without adjuvant chemo - RFS in ctDNA+	Study closed
SAGITTARIUS	Colon high-risk stage II and stage III	ctDNA-guided group versus standard group	—	700	—	Not available
CIRCULATE-AIO^73^	Colon stage II	Chemo or no chemo in post-op ctDNA+ patients	Not reported	4812	DFS	Not available
CIRCULATE 2-PRODIGE 70^74^	Colon stage II	Chemo or no chemo in post-op ctDNA+ patients	ddPCR for *WIF1* and *NPY* methylation markers	1980	DFS	Not available
PEGASUS^75^^,^^76^	Colon T4N0 and stage III	Escalated or de-escalated treatment based on ctDNA monitoring	LUNAR1®	140	MRD after surgery and chemotherapy	Feasibility completed 7% of recurrences in ctDNA− mainly in patients with lung or peritoneal recurrences 46% of patients switched to FOLFIRI at 3 months experienced ctDNA clearance
GALAXY^77^, ^78^, ^79^, ^80^	Colorectal stage II-III and resectable stage IV	—	Signatera®	1040	Association between post-operative ctDNA and recurrence risk	ctDNA+ patients after surgery had an inferior DFS (HR 10.53) ctDNA clearance after ACT was associated with better response and better DFS (HR 6.72)
VEGA^77^	Colorectal stage II-III and resectable stage IV	Chemo or no chemo in post-op ctDNA− patients	Signatera	1240	DFS	Not available
ALTAIR^77^	Colorectal stage II-III and resectable stage IV	Trifluridine/tipiracil or placebo in ctDNA+ patients	Signatera	240	DFS	Not available
CIRCULATE-PAC PRODIGE 88 (EU 2023-505046_26-00)	Colorectal stage II-III	FOLFIRI versus trifluridine tipiracil versus surveillance in patients with ctDNA+ after adjuvant chemotherapy with FOLFOX/CAPOX	MethylationddPCR	1680	PFS	Not available
ERASE-CRC^81^	Colon stage III and high-risk stage II	First: FOLFOX or FOLFOXIRI in post-op ctDNA+ Second: trifluridine/tipiracil or no treatment in post-adjuvant ctDNA+	Not reported	300	ctDNA clearance after adjuvant and post-adjuvant chemo	Not available
SU2C ACT3^82^	Colon stage III	First: FOLFIRI Second: surveillance Third: targeted therapy	Sequencing panel (Reveal, Guardant Health)	73	ctDNA clearance 1 month after additional chemotherapy	13.7% were ctDNA+ after adjuvant chemo 90% of ctDNA+ had no actionable biomarker
IMRROVE-IT2^83^	Colorectal high-risk stage II and III	ctDNA approach versus standard imaging for follow-up	NGS and ddPCR	250	The proportion of patients receiving curative-intended resection or local metastasis-directed treatment	Not available
BESPOKE^84^^,^^85^	Colorectal, stage I-IV	—	Signatera	2000	Impact of ctDNA on adjuvant treatment decision Determine rate of recurrence of asymptomatic patients based on ctDNA	Post-operative ctDNA positivity was associated with worse DFS (HR 20.8) Better DFS in ctDNA+ with ACT compared to observation (HR 3.9) No benefit of ACT in ctDNA− (HR 1.1) ctDNA clearance with ACT and ctDNA status during surveillance were prognostic of patient outcome

ACT, adjuvant chemotherapy; ctDNA, circulating tumor DNA; DFS, disease-free survival; ddPCR, droplet digital polymerase chain reaction; HR, hazard ratio; MRD, minimal residual disease; NGS, next-generation sequencing; RFS, recurrence-free survival.

A prospective UK multicenter prospective trial (TRACC) is recruiting patients with resected stage II and III CRC to study the non-inferiority of a ctDNA-guided treatment to standard of care, with a view to avoiding unnecessary adjuvant chemotherapy. Analysis of MRD detection by ctDNA showed that 93.4% of patients had detectable ctDNA before surgery and 13% were MRD-positive after surgery. 42.9% of ctDNA-positive patients relapsed while only 8.6% of ctDNA-negative patients did. In the multivariable analysis, ctDNA status was the most significant prognostic factor associated with RFS. The results of this trial are still awaited^69^^,^^70^ (Table 2) (Figure 3A).

In the MEDOCC-CrEATE trial, dedicated to stage II colon cancer, patients are randomized to pursue or not a ctDNA-guided adjuvant chemotherapy. In the ctDNA-guided group, patients with positive ctDNA will receive eight cycles of CAPOX (capecitabine + oxaliplatin), while patients with negative ctDNA will receive the same treatment modality as patients in the control group^71^ (Table 2). A very similar design was chosen for the US COBRA trial, which examined ctDNA clearance after adjuvant chemotherapy and RFS in patients in the ctDNA-positive arm^72^ (Table 2) (Figure 3A). However, the latter trial was recently closed to enrollment due to an unexpected rate of false-positive results observed in the planned interim analysis with the tumor-agnostic test used in this study.

SAGITTARIUS is a randomized controlled trial that will recruit 700 patients with high-risk stage II or stage III colon cancer across 25 centers in three European countries. The aim of this trial is to compare the standard-of-care treatment to a personalized care strategy based on the presence or absence of MRD 4 weeks after surgery. The alternative approaches include chemotherapy, targeted therapy, immunotherapy, and surveillance (Figure 3A).

On the other hand, various clinical trials have adopted another type of study design, consisting in testing ctDNA in all patients and comparing different treatment options in both ctDNA-positive and -negative patients, in other words trials ‘testing the treatment’ (Figure 3B).

The large German/Austrian/Swiss CIRCULATE-AIO and French/Belgium CIRCULATE 2-PRODIGE 70 trials are randomizing patients with stage II resected colon cancer with post-operative positive ctDNA to receive or not adjuvant chemotherapy. The primary endpoint is DFS in both studies, which share a very similar design^73^^,^^74^ (Table 2) (Figure 3B). In the German trial, an academic tumor-informed assay will be used as a methylation tumor-agnostic assay that is currently used in the French trial. However, the French CIRCULATE 2-PRODIGE 70 trial has been recently amended to integrate a tumor-informed commercial test (SIGNATERA®) in addition to the original methylation test.

The PEGASUS study is designed for patients with resected stage III and T4N0 stage II colon cancer, evaluating an escalation or de-escalation adjuvant treatment based on ctDNA status monitored every 3 months^75^ (Table 2). Initial results were presented at ESMO 2023 and ASCO GI 2024 with a median follow-up of 21.2 months. They showed the feasibility of this ctDNA-based approach and that 7% of ctDNA-negative patients relapsed despite treatment de-escalation, with most disease relapses observed in the peritoneum and lung in this group, both sites known to be associated with low release of ctDNA into the bloodstream. In addition, 46% of patients with a positive ctDNA test 3 months after CAPOX chemotherapy that were switched to FOLFIRI [5-fluorouracil (5-FU) + irinotecan] experienced ctDNA clearance, which seems to be a promising signal^76^ (Figure 3B).

One of the largest trials in this field is the CIRCULATE-Japan platform. This platform is composed of three parallel studies—GALAXY, VEGA, and ALTAIR—which are investigating the benefit of ctDNA detection in patients with resectable stage II-IV CRC. GALAXY is an observational study dedicated to ctDNA monitoring. The VEGA trial is evaluating a de-escalation approach in patients without detectable ctDNA 4 weeks after surgery. Patients are randomized to receive or not 3 months of adjuvant CAPOX. The ALTAIR study is designed to establish the superiority of trifluridine/tipiracil to placebo in patients with positive ctDNA at any time within 2 years after surgery. Preliminary results from the GALAXY study have been recently published. With a median follow-up of 24 months, patients with positive ctDNA during the MRD window (2-10 weeks after surgery or before chemotherapy) had a shorter DFS (HR 10.53). At 24 months, the DFS was 28.9% in ctDNA-positive patients compared with 85.9% in ctDNA-negative patients. ctDNA clearance after adjuvant chemotherapy was an indicator of response and was associated with a better DFS (HR 6.72)^77^, ^78^, ^79^, ^80^ (Table 2) (Figure 3B).

Another prospective multicenter study is the French PRODIGE 88 trial which will randomize patients with resected high-risk stage II or III disease and with a positive ctDNA 3-6 months after chemotherapy cessation to receive FOLFIRI or trifluridine/tipiracil or to be followed up, with an endpoint of DFS (EU 2023-505046_26-00). A similar trial (ERASE-CRC) including patients with resected stage III and high-risk stage II colon cancer with post-operative positive ctDNA randomizes patients to receive FOLFOX/CAPOX versus FOLFOXIRI (5-FU+ oxaliplatin + irinotecan) in the first part. In the second part, patients with positive ctDNA at the end of chemotherapy are randomized to receive or not trifluridine/tipiracil for six cycles. The primary endpoint is ctDNA clearance after adjuvant and post-adjuvant chemotherapy^81^ (Table 2) (Figure 3B).

SU2C ACT3 clinical trial is enrolling patients with stage III colon cancer treated with adjuvant chemotherapy. ctDNA is analyzed 3-6 weeks after adjuvant chemotherapy. ctDNA-negative patients undergo surveillance. ctDNA-positive patients who are biomarker-negative are randomized 1 : 1 to treatment with FOLFIRI or surveillance. ctDNA-positive patients who are biomarker-positive are treated with biomarker-targeted therapy for 6 months. The primary endpoint is ctDNA clearance 1 month after completion of additional chemotherapy in ctDNA-positive patients who are biomarker-negative. To date, 73 patients have been enrolled. 13.7% were ctDNA-positive and 90% of them did not have a targetable biomarker.^82^

Finally, the role of ctDNA in post-operative surveillance is evaluated in the Danish IMPROVE-IT2 study. A ctDNA-guided surveillance approach is compared to standard radiologic follow-up. The primary endpoint is the proportion of patients receiving curative-intended resection or local metastasis-directed treatment^83^ (Table 2). The BESPOKE study is another ongoing prospective observational trial evaluating the role of ctDNA in adjuvant treatment decision making and in the detection of disease recurrence in stage I-IV CRC. At 24.8 months, post-operative ctDNA positivity was associated with worse DFS (HR 20.8). Patients with positive ctDNA had better DFS with chemotherapy compared to surveillance (HR 3.9) and no benefit from adjuvant chemotherapy was observed in ctDNA-negative patients. ctDNA status after adjuvant chemotherapy and during follow-up was prognostic of patient outcome^84^^,^^85^ (Table 2).

### Non-metastatic rectal cancer

Preoperative chemoradiation or radiotherapy followed by total mesorectal excision surgery (TME) remains the standard of care for the treatment of stage II and III rectal adenocarcinoma.^86^ Total neoadjuvant treatment (TNT), evaluated in several clinical trials, was associated with improved local response and pathologic complete response (pCR) with a benefit in terms of OS only in the PRODIGE 23 trial.^87^, ^88^, ^89^

Different studies have evaluated the clinical utility of ctDNA in predicting response to neoadjuvant treatment and detecting disease recurrence after surgery in locally advanced rectal cancer (LARC).

The prognostic value of baseline ctDNA assessed by Appelt et al. showed that patients with positive ctDNA (methylation test) at baseline had worse 5-year OS and more distant metastases at 5 years in comparison to ctDNA-negative patients^90^ (Table 3). In a cohort study of 36 LARC patients treated with neoadjuvant chemoradiation (NCRT) followed by surgery, pretreatment ctDNA positivity was associated with reduced post-operative DFS and OS by 1.47 and 1.41 years, respectively^91^ (Table 3).

**Table 3:** Studies evaluating the role of ctDNA in predicting response to neoadjuvant chemoradiation and disease relapse after surgery in non-metastatic rectal cancer

Study	ctDNA analysis	Sample size	Primary endpoint	ctDNA detectionrate	Results
Tie et al.^96^	Safe-SeqS	159	Role of ctDNA in risk stratification	Baseline: 77% Preop: 8.3% Post-op: 12%	- No difference in RFS based on baseline ctDNA - Inferior RFS in ctDNA+ after NCRT (*P* < 0.001) and after surgery (*P* < 0.001) - Post-op ctDNA associated with risk of recurrence irrespective of adjuvant chemo - ctDNA was not predictive of pCR
Appelt et al.^90^	NGS for *NPY* hypermethylation	146	OS and distant metastases rate	Baseline: 20.5%	- Worse 5-year OS in positive meth DNA (47% versus 69% HR 2.08) - Lower metastasis rate at 5 years (55% versus 72% HR 2.20)
Pazdirek et al.^91^	BEAMING	36	Association between ctDNA changes in early days of NCRT and therapy outcome	Preop: 21.2%	- Pretreatment ctDNA+ was associated with a median reduction of RFS (1.47 years) and OS (1.41 years) (*P* = 0.015, *P* = 0.010) - Reduction or elimination of ctDNA in all patients with no correlation with TRG or TNM
Murahashi et al.^92^	NGS	85	ctDNA correlation with pathological response to preop therapy and post-op recurrence	Baseline: 57.6% Preop: 22.3%	- ctDNA predictor of response to preop therapy - Post-op ctDNA prognostic of recurrence with cumulative effect on RFS
McDuff et al.^93^	NGS	29	Ability of ctDNA to predict surgical outcome or recurrence after NCRT	Preop: 34.6%	- Higher rate of R0 in preop ctDNA− than ctDNA+ (88% versus 44%, *P* = 0.028) - Higher recurrence rate in post-op ctDNA+ versus 13% in ctDNA− (NPV 87%) - Worse RFS in ctDNA+ (HR 11.6) (*P* = 0.007)
Zhou et al.^94^	NGS	106	Role of ctDNA in predicting tumor response to NCRT, tumor burden and prognosis	Preop at four time points: 75%, 15.6%, 10.5%, and 6.7%	- ctDNA associated with shorter MFS (*P* < 0.05) - Median VAF of mutations in baseline ctDNA was predictor of MFS (*P* < 0.001)
GEMCAD 1402^95^	NGS	180	Ability of ctDNA to predict response, recurrence, and survival after TNT	Baseline: 83% Preop: 15%	High risk of recurrence, short DFS (*P* = 0.033) and OS (*P* < 0.0001) in post-op ctDNA+ but no correlation with pathologic response
DYNAMIC RECTAL^97^	—	230	The use of adjuvant chemo after TNT and TME (ctDNA-guided versus standard strategy)	Post-op: 28%	- 46% of patients in the ctDNA-guided group received chemotherapy compared to 77% in the standard group - The 3-year RFS: 76% for ctDNA-guided group compared to 82% for the standard-of-care group
Wang et al.^98^	NGS	119	Role of a risk model combining ctDNA and MRI to detect pCR	Baseline: 84%	- ctDNA clearance during NCRT was correlated with pTRG (95.7% in pTRG 0, 77.8% in pTRG1, 71.1% in pTRG2, and 66.7% in pTRG3, *P* = 0.008) and was associated with low probability of non-pCR (OR = 0.11) - The detection of acquired mutations was 3.8%, 8.3%, 19.2%, and 23.1% in pTRG 0, 1, 2, and 3 groups, respectively (*P* = 0.02) - A risk score combining ctDNA and mrTRG had a higher pCR prediction performance than ctDNA or mrTRG alone - Patients with detectable driver mutations and high-risk features after surgery had the highest recurrence risk (*P* < 0.001)

ctDNA, circulating tumor DNA; HR, hazard ratio; MFS, metastasis-free survival; MRI, magnetic resonance imaging; mrTRG, MRI tumor regression grade; NGS, next-generation sequencing; NPV, negative predictive value; NRCT, neoadjuvant chemoradiation therapy; OS, overall survival; pCR, pathologic complete response; post-op, post-operative; pTRG, pathologic tumor regression grade; preop, preoperative; RFS, recurrence-free survival; TME, total mesorectal excision; TNM, tumor nodes metastases; TNT, total neoadjuvant treatment; TRG, tumor regression grade; VAF, variant allele frequency.

The correlation between ctDNA after neoadjuvant treatment or after TME and patient outcome has been demonstrated in various studies.^92^, ^93^, ^94^ Serial ctDNA analysis in 85 patients with LARC showed that changes between baseline and post-neoadjuvant treatment ctDNA status were an independent predictor of pathological response to preoperative treatment. Post-operative ctDNA detection was also a prognostic factor for disease recurrence. A combined analysis of post-operative CEA and ctDNA revealed cumulative effects on RFS^92^ (Table 3).

Another similar prospective study with 29 patients demonstrated a higher rate of R0 resection in patients with undetectable preoperative ctDNA compared to patients with positive ctDNA. In this study, after a median follow-up of 20 months, all patients who had positive post-operative ctDNA experienced recurrence, whereas only 2 out of 15 patients with undetectable post-operative ctDNA experienced recurrence (negative predictive value: 87%), and post-operative ctDNA positivity was associated with worse DFS (HR 11.56)^93^ (Table 3). Zhou et al. included 106 patients with LARC. ctDNA was determined at four time points before surgery. All patients who had pCR had preoperative negative ctDNA. In addition, positive ctDNA was associated with a shorter metastasis-free survival (MFS)^94^ (Table 3).

Further larger studies gave similar results regarding the prognostic role of ctDNA in LARC. GEMCAD 1402 is a randomized phase II multicenter clinical trial including patients with LARC treated with TNT (mFOLFOX ± aflibercept followed by chemoradiation). ctDNA was measured at baseline, after TNT within 48 h preoperatively. ctDNA was detectable in 83% of patients at baseline and in 15% after TNT. Patients who had detectable pre-surgery ctDNA had a higher risk of recurrence, and shorter DFS (HR 4) and OS (HR 23), but no association was found between ctDNA and pathological response^95^ (Table 3). The GEMCAD REVEAL study is an ongoing prospective trial that aims to evaluate the role of ctDNA as a predictive marker of relapse in patients treated with TNT followed by TME or surveillance depending on the clinical assessment of local response (NCT05674422).

Tie et al. also showed a worse RFS in ctDNA-positive patients after NCRT (HR 6.6) and after surgery (HR 13.0) in their prospective study. Post-operative ctDNA positivity was associated with high risk of recurrence irrespective of adjuvant chemotherapy. However, here again ctDNA was not predictive of pCR^96^ (Table 3). Based on these data, DYNAMIC RECTAL is an ongoing randomized controlled trial to determine the role of ctDNA-guided adjuvant treatment decision making in LARC. Eligible patients have LARC treated by TNT followed by TME and are fit for adjuvant chemotherapy. Patients are randomized 2 : 1 to a standard of care or ctDNA-guided approach. ctDNA-positive patients are treated with 4 months of chemotherapy and ctDNA-negative are observed if ypN0 or treated according to the clinician’s choice if ypN+. The primary endpoint is adjuvant chemotherapy use. Two hundred and thirty patients have been included to date. At a median follow-up of 37 months, 46% of patients in the ctDNA-guided group received chemotherapy compared to 77% of patients in the standard group. The 3-year RFS was 76% for the ctDNA-guided group compared to 82% for the standard-of-care group.^97^

Though ctDNA clearance alone does not seem strongly correlated with pCR after NCRT, Wang et al. created a risk model combining ctDNA and magnetic resonance imaging (MRI) to detect pCR. ctDNA was measured before NCRT, at the 15th and the 25th fractions of NCRT, 0- 1 days before surgery and 5-12 days after surgery. ctDNA clearance during NCRT was associated with low probability of non-pCR. A risk score predictive model combining both ctDNA and MRI tumor regression grade (mrTRG) had a higher pCR/non-pCR prediction performance compared with models derived from only ctDNA or only mrTRG. The detection of potential CRC driver gene mutations was associated with worse RFS (HR 9.29). Patients who had detectable driver mutations and high-risk features after surgery had the highest recurrence risk (HR 90.29)^98^ (Table 3).

Based on these interesting preliminary results, ctDNA could be a potent marker for a personalized treatment approach to LARC. The role of ctDNA in predicting response to neoadjuvant treatment could help in the future to achieve an organ preservation strategy for the most suitable patients.

### Resected metastatic colorectal cancer

Complete surgical resection of all metastatic sites remains the cornerstone of a curative-intent approach to achieving long-term survival.^99^ This approach combines surgical resection and local ablation with perioperative systemic chemotherapy and locoregional treatments in selected patients. Nevertheless, standard peri- and post-operative chemotherapy has failed to increase OS in phase III randomized controlled trials, and disease recurrence occurs in more than two-thirds of cases despite post-operative chemotherapy.^100^, ^101^, ^102^ Several clinical risk scores have been developed to predict the risk of recurrence in patients with colorectal liver metastases (CRLM), but their accuracy in helping to make individual treatment decisions remains poor.^103^, ^104^, ^105^ Therefore, the current tools are insufficient to properly select good candidates for a potentially curative strategy for CRLM (and other metastatic sites) and a post-operative oxaliplatin-based regimen seems unable to improve OS in unselected patients.^106^

The clinical validity of ctDNA in detection of MRD after curative-intent surgery in CRLM patients has been assessed in several small-cohort studies, with different ctDNA detection methods. During the post-operative period, ctDNA remains present in 14%-80% of patients in the early post-operative period and in 28%-44% after post-operative chemotherapy^11^^,^^34^^,^^107^, ^108^, ^109^ (Table 4). A multicenter study including 47 patients with mCRC who underwent resection of metachronous or synchronous liver metastases and were suitable for ctDNA monitoring reported the following results: 93% of patients who had an R0 resection were ctDNA-negative post-operatively, while all patients with R2 resection were ctDNA-positive. In this study, all patients with positive ctDNA experienced disease recurrence^110^ (Table 4). Several prospective studies confirmed the predictive value of post-operative ctDNA for disease recurrence, with HRs ranging from 4.9 to 13^108^^,^^111^, ^112^, ^113^, ^114^ (Table 4).

**Table 4:** Clinical trials evaluating the role of ctDNA as a predictive and prognostic marker in fully resected metastatic colorectal cancer

Trial	ctDNA analysis	Sample size	Metastatic site	Objectives	ctDNA detection rate	Results
Diehl et al.^107^	BEAMING	18	—	Role of ctDNA in disease monitoring	—	- The change in post-op ctDNA was correlated with the extent of surgical resection - Post-op ctDNA+ relapsed within 1 year
Schøler et al.^34^	ddPCR	23	—	Correlation between post-op ctDNA and risk of relapse	—	3 months post-op ctDNA+ was associated with high risk of relapse (HR 4.9)
Benešová et al.^110^	PCR	47	Liver	Correlation between post-op ctDNA, resection rate, and disease recurrence	Preop: 100% Post-op: 7% (R0), 57% (R1) and 100% (R2)	- 100% of recurrence within 7 months in case of post-op ctDNA+ in R0/R1 resected patients - 100% of ctDNA+ in case of recurrence
PRODIGE-14^120^	ddPCR	153	Liver	ctDNA correlation with resection rate and OS	Baseline: 91%	- ctDNA+ at 4 weeks during preop chemotherapy was associated with lower R0/R1 resection - Preop ctDNA+ in R0/R1 was associated with shorter OS
Boysen et al.^108^	ddPCR, MassARRAY®	35	Liver Lung	ctDNA correlation with disease recurrence after local therapy for lung/liver metastases	24%	- 49% of patients with positive ctDNA developed recurrence - Post-op ctDNA+ status was correlated with recurrence (HR 7.5)
He et al.^121^	NGS	20	Liver	Perioperative ctDNA correlation with tumor burden and disease recurrence	Preop: 85%	- Preop ctDNA predictive of relapse - Low preop ctDNA associated with prolonged PFS
Tie et al.^109^	Safe-SeqS	61	Liver	Prognostic impact of post-op ctDNA	Baseline: 85% Post-op: 24.4%	- Post-op ctDNA+ associated with worse PFS (HR 6.3) and OS (HR 4.2) - 0% 5-year RFS in end-of-treatment ctDNA+ versus 75.6% in ctDNA− (HR 14.9) - ctDNA significantly decreased (40-fold) during neoadjuvant chemo but was not associated with RFS
Wang et al.^11^	NGS	91	Liver	Clinical value of serial ctDNA analysis in predicting clinical outcome	Baseline: 88.7% (median VAF 21.42%) Post-op: 41% (median VAF 0%) Post-ACT: 44.9% After progression: 95.5%	- Higher VAF in baseline ctDNA associated with high tumor burden - ctDNA clearance associated with better tumor response - Post-op and post-ACT associated with worse RFS - Higher recurrence rate in post-op ctDNA+ (79.4% versus 41.7%) and post-ACT ctDNA+ (77.3% versus 40.7%) - Decreased post-ACT VAF associated with lower recurrence rate (63.6% versus 92.3%)
Bolhuis et al.^111^	ddPCR	23	Liver	Association between ctDNA, pathologic response, and RFS	Baseline: 78% Post-op: 26%	- Post-op ctDNA was correlated with recurrence (100% ctDNA+ versus 65% ctDNA−) and RFS (4.8 months in ctDNA+ versus 12.1 monthsin ctDNA−) - Post-op ctDNA strongly correlated with pathological response (100% post-op ctDNA–had TRG 1-3) - Post-op ctDNA (HR 3.3) and pathologic non-response (HR 4.6) were associated with poorer RFS
Loupakis et al.^112^	Bespoke multiple PCR, NGS	112	Liver Lung Peritoneum Others	Ability of ctDNA to detect MRD and association with DFS and OS	Post-op: 54.4%	- Post-op ctDNA was associated with DFS (HR 5.8) and OS (HR 16.0) - Post-op ctDNA was the most significant prognostic factor associated with DFS (HR 5.78)
Kobayashi et al.^117^	Guardant360®	40	Liver	Correlation between preop ctDNA and recurrence risk after surgery	Preop: 80%	- Only one patient with preop ctDNA− had recurrence - Preop ctDNA+ was correlated with RFS (HR 7.6) and OS (HR not available)
Nimeiri et al. ^113^	NGS	69	Liver Lung Peritoneum Others	Prognostic value of ctDNA	Post-op: 54%	- MRD+ was associated with lower DFS (HR 4.97) and OS (HR 27.05) - ctDNA+ during follow-up was associated with lower DFS (HR 8.78) and OS (HR 20.06)
Nishioka et al.^114^	NGS	105	Liver	Effect of co-mutation of RAS and TP53 on post-op ctDNA and risk of recurrence	Post-op: 30%	- Multiple liver metastases and co-mutated RAS/TP53were independently associated with post-op ctDNA - Post-op ctDNA+ (HR 2.04) and extrahepatic disease (HR 2.45) were independently associated with worse RFS - ctDNA+ within 180 days was the only independent factor for recurrence
Reinert et al.^115^	ddPCR	115	Liver	Value of serial ctDNA analysis for post-op prognosis and guidance of CT imaging	Preop: 86.4% Post-op: 32.5%	- Stratification of patients at low and high risk after surgery depending on ctDNA (HR 7.6; HR 4.3 respectively), with a PPV of 100% - ctDNA+ was the only marker of relapse after surgery - All patients with ctDNA+ relapsed versus 42.6% in ctDNA– - Recurrence diagnoses in patients with undetermined CT were delayed by a median 2.8 months
Øgaard et al.^116^	ddPCR	96	Liver	Role of serial ctDNA analysis in post-op surveillance	Preop: 97.6% Post-op: 65%	- Post-op (HR 4.5) and post-ACT (H 8.4) was associated with RFS (PPV 100%) - ctDNA growth rate significantly correlated with poor OS (HR 1.6) - 3.1 months median time to radiologic recurrence
Liu et al.^118^	NGS	134	Liver	Role of post-op ctDNA to predict survival and recurrence	Post-op: 31.3%	- Shorter DFS in ctDNA+ (HR 2.96) - Shorter DFS in patients with higher AFs (HR 1.98) - Longer DFS in ctDNA+ who received adjuvant chemo for >2 months (HR 0.377)

ACT, adjuvant chemotherapy; AFs, allele frequencies; CT, computed tomography; ctDNA, circulating tumor DNA; ddPCR, droplet digital polymerase chain reaction; DFS, disease-free survival; HR, hazard ratio; MRD, minimal residual disease; NGS, next-generation sequencing; OR, odds ratio; OS, overall survival; PFS, progression-free survival; post-op, post-operative; PPV, positive predictive value; preop, preoperative; RFS, recurrence-free survival; TRG, tumor regression grade; VAF, variant allele frequency.

Moreover, ctDNA status was able to detect recurrence earlier than standard work-up using conventional surveillance programs with a median interval of 9 months with a positive predictive value of 100%^115^^,^^116^ (Table 4).

Two recent prospective cohorts, including 54 and 91 patients, respectively, showed that surgery can induce ctDNA clearance in >50% of ctDNA-positive patients preoperatively (from 87%-88% to 31%-41%). Moreover, post-adjuvant chemotherapy ctDNA status demonstrated, as expected, a better correlation with post-operative recurrence compared to the early post-operative period, with 0% 5-year RFS in ctDNA-positive patients compared with 75.6% in ctDNA-negative patients at the end of treatment (HR 14.9)^11,109^ (Table 4). The relevance of ctDNA status in terms of post-operative relapse has also been confirmed in 40 patients with solitary CRLM by Kobayashi et al.,^117^ suggesting that ctDNA is an interesting selection tool for surgery (Table 4). The quantitative aspect of ctDNA in terms of median allele frequency assessed after curative resection of CRLM has also been evaluated in one study and correlated significantly with DFS in 134 patients^118^ (Table 4).

Beyond recurrence, the association between MRD and OS represents a key point in terms of clinical benefit for patients with CRLM, mainly in the case of potentially resectable disease eligible for extensive surgery. A positive correlation between negative ctDNA and OS was demonstrated in some of these studies with a lower magnitude effect compared to the correlation observed between ctDNA and disease recurrence^113^^,^^114^^,^^116^ (Table 4). A recent meta-analysis including 2868 patients with mCRC undergoing locoregional treatments (including complete surgery, local ablation, and cytoreductive surgery) confirmed the predictive and prognostic value of pre-and/or post-operative ctDNA for both PFS and OS.^119^

However, no large study has directly compared ctDNA baseline status and dynamic changes with other validated prognostic markers (clinical, laboratory, radiological, and pathological) in mCRC. Several large prospective ongoing cohorts will assess in detail the added value of ctDNA in potentially resectable mCRC (NCT05787197, NCT03189576, NCT04704960, NCT04752930).

In addition to predicting disease recurrence and OS, many other studies have tested the role of ctDNA in predicting response to neoadjuvant treatment in order to select the patients most suitable for liver resection. Moreover, post-adjuvant treatment ctDNA could also predict early disease recurrence. The PRODIGE-14 trial included 153 patients with mCRC with potentially resectable liver metastases treated with chemotherapy. Circulating tumor cells and ctDNA were assessed at inclusion, after 4 weeks of treatment and before liver metastasis surgery. ctDNA detection sensitivity at baseline was 91% and decreased during treatment. Persistently detectable ctDNA at 4 weeks was associated with a lower R0/R1 liver metastases resection rate^120^ (Table 4). He et al. demonstrated that preoperative ctDNA was predictive of disease recurrence and that no preoperative ctDNA was associated with prolonged DFS^121^ (Table 4).

Tie et al. enrolled 61 patients with resectable CRC liver metastases in a prospective two-arm cohort. Cohort 1 was planned for liver resection followed by adjuvant chemotherapy for up to 6 months. Cohort 2 was planned to receive neoadjuvant chemotherapy followed by liver resection followed by 3-4 months of adjuvant chemotherapy. In cohort 2, there was a major decrease in ctDNA during neoadjuvant chemotherapy, but no association with better RFS. Positive ctDNA after surgery was associated with worse RFS (HR 6.3) and OS (HR 4.2)^106^ (Table 4).

All these trials reported the potential clinical utility of ctDNA in guiding treatment decision making in resectable mCRC. By assessing the response to systemic treatment, ctDNA could help to select patients for resection of metastases. ctDNA after surgery could allow detection of MRD and selection of patients for adjuvant treatment. In addition, serial ctDNA analyses after surgery and adjuvant treatment could detect early disease recurrence with high sensitivity compared with the standard surveillance approach. Large randomized clinical trials should now be conducted to validate these promising preliminary results.

## Conclusions

Analysis of ctDNA is a revolutionary non-invasive approach that has rapidly emerged and progressed to affect management of all types of cancer including CRC. Through all the studies presented in this review, we can see that ctDNA analysis has an important place in guiding the best therapeutic strategy for patients with resectable non-metastatic CRC or mCRC. While the undeniable role of ctDNA as an independent prognostic factor for RFS and OS seems clear, its integration into the current treatment decision-making process, alone or in combination with more usual laboratory and pathological criteria, remains to be fully defined. The choice of method for detecting ctDNA is, however, a very important issue that needs to be addressed and tailored to each situation in the future, with the different approaches having respective advantages and disadvantages. These methods will thus need to be chosen adequately for optimal management of our patients. Even though ctDNA is not today the perfect tool for selecting patients for optimal personalized treatment strategies, it represents a major advance in this field. A very large number of therapeutic trials are currently testing the added value of ctDNA in our treatment decisions and should help to answer these questions in the coming years.
